# Availability of outpatient methadone maintenance

**DOI:** 10.1186/1940-0640-10-S1-A8

**Published:** 2015-02-20

**Authors:** Timothy B Creedon, Amity E Quinn, Xiaodong Liu, Dominic Hodgkin, Constance M Horgan

**Affiliations:** 1 The Heller School for Social Policy and Management, Brandeis University, Waltham, MA, 02454, USA; 2 Department of Psychology, Brandeis University, Waltham, MA, 02454, USA

## Background

Multiple forms of effective treatment for opioid use disorders (OUDs) exist, but they have remained in short supply and have been underutilized where available. Simultaneously, OUDs persist as a large and growing public health problem across the United States, leading to epidemic levels of overdose death as well as many other damaging societal consequences. Our goal was to assess the current state of the U.S. substance abuse treatment system and measure its capacity for treating OUDs. Focusing on outpatient methadone maintenance therapy (OPMM), an effective but less frequently studied treatment in recent years, we investigated two primary questions about privately-run substance abuse treatment facilities: 1) What facility-level characteristics best predicted the provision of OPMM? 2) How much of the variation in OPMM availability was attributable to differences between states?

## Methods

We conducted a cross-sectional, secondary analysis of facility-level data from the 2012 National Survey of Substance Abuse Treatment Services. Our analysis focused on privately run facilities located in the 50 states and the District of Columbia. The outcome of interest was provision of OPMM. Covariates of interest were facility characteristics (ownership type, hospital status, payment sources, treatment focus, language, religious affiliation, and admissions levels) and a set of contextual measures including level of urbanization, state, and region. We generated descriptive univariate and bivariate statistics to elucidate facility characteristics and the relationships between them. We employed multilevel mixed-effects logistic regression models to investigate our research questions, while accounting for clustering of facilities within states.

## Results

Out of a sample of 10739 facilities, about 10 percent reported offering OPMM. Accepting Medicaid (OR = 2.56; SE = 0.62), working in other languages in addition to English (OR = 2.05; SE = 0.16), and operating in the Northeast (OR = 2.95; SE = 0.86) were among the strongest predictors of offering OPMM. Nonprofit facilities not affiliated with hospitals (OR = 0.14; SE = 0.01) and those situated in rural areas (OR = 0.34; SE = 0.07) were among the least likely to provide OPMM. Conditional on model fixed effects, about 21 percent of variation in the likelihood of offering OPMM could be attributed to differences between states (SE = 0.057). Associations between OPMM and Medicaid acceptance also varied considerably from state to state (*τ* = 2.01; SE = 0.62).

## Conclusions

Very few facilities offered OPMM in 2012, consistent with trends observed over the previous decade. Most facility characteristics examined in this study were strong independent predictors of OPMM availability. Even so, OPMM availability continued to vary widely between states. These patterns have important policy implications. By examining differences between facilities that are most and least likely to offer OPMM, it may be possible to identify key catalysts and obstacles that could alternatively support and thwart further implementation of life-saving services like OPMM. Persisting state, regional, and urban/rural variations suggesting external, contextual dynamics—like resource availability and political climate—also need to be addressed. Therefore, the findings of this study may aid in the development of a more targeted, efficient strategy for expanding access to OPMM.

